# Beyond Individualism and Independence: Exploring Collectivism and Interdependence as Paradigms of Healthcare and Health Professions Education

**DOI:** 10.5334/pme.2000

**Published:** 2025-12-04

**Authors:** Eusang Ahn, Stefanie S. Sebok-Syer, Jerry M. Maniate, Kaitlin Endres, Warren J. Cheung

**Affiliations:** 1 Department of Emergency Medicine, University of Ottawa, Ottawa, Ontario, Canada; 2 Department of Emergency Medicine, Stanford University, Stanford, California, USA; 3 Departments of Medicine and Innovation in Medical Education, University of Ottawa, Canada; 4 Bruyère Health Research Institute, Ottawa, Ontario, Canada

## Abstract

**Purpose:**

Healthcare is delivered by teams, yet individualism continues to be foregrounded in training and assessments. In contrast to individualism is collectivism, a sociocultural phenomenological lens that considers the degree to which people are integrated into groups. Along with collectivism is interdependence, which characterizes how two or more people or things are interconnected with each other. The purpose of this review was to examine the current body of work on collectivism and interdependence to examine their potential within healthcare and health professions education (HPE).

**Method:**

This narrative review included English-language publications from peer-reviewed journals between 1990 and 2024, and excluded non-English articles, perspective pieces, commentaries, and studies that were unrelated to healthcare or HPE. The initial search in January 2024 included MEDLINE, Embase, PsycINFO and ERIC databases and was updated in November 2024 with forward and backward reference mining.

**Results:**

In total, 3,415 articles were identified, of which 63 were included in the review. We used reflexive thematic analysis to consolidate the literature. Our analysis developed four interconnected themes: 1) reshaping the culture of healthcare and the learning climate of HPE, 2) accounting for individual contributions to the team, 3) the many facets of leadership, and 4) belongingness as an essential step towards collective well-being. Together, these themes expose tensions between individualistic educational structures and the team-based realities of clinical work, and highlight pathways toward relational, trust-based learning environments.

**Conclusion:**

Healthcare and HPE can be effectively examined through the paradigms of collectivism and interdependence, which transcend and challenge the current models of individualism and independence. Collectivism and interdependence offer conceptual and practical tools to reorient HPE toward relational accountability, shared leadership, and compassionate belonging. By embracing these guiding principles, healthcare workers and educators can better prepare for the collaborative, team-based nature of healthcare, and create protective and transformative learning environments.

## Introduction

Within healthcare and health professions education (HPE), we select, train, assess, license, hire, pay, regulate, and remediate *individuals* [1]. This approach is problematic because patient care is frequently delivered by teams, which includes the constellation of complex interpersonal relationships, competing interests, communication challenges, and cultural factors inherent to teamwork. For instance, Lingard describes how adverse events in homecare, often exceeding 10% among seniors, stem not from individual incompetence, but from breakdowns in collective coordination across providers [1]. Examples such as these illustrate some of the limits of individualist models of competence and necessitate that we consider alternative conceptualizations.

Medicine’s focus on individualism can be traced back to resistance against collectivist educational reform rooted in a mid-nineteenth century Protestant-Capitalist tradition, producing a “lingering, historically conditioned pattern of heroic individualism” [2]. Despite the absence of strong evidence to support the progressive independence within many HPE systems [3], independence persists as the ultimate goal of training in various HPE curricula [4,5,6]. Therefore, there is an urgent need to address this discordance between how learners are trained and how healthcare is delivered and we believe one approach is to rethink the foundational paradigms that exist within healthcare and HPE.

Collectivism and interdependence offer valuable lenses through which to re-examine learning culture, leadership, assessment, and identity formation. Collectivism is a sociocultural phenomenological lens that exists on a continuum with individualism [7]. While individualist societies emphasize personal achievements and individual rights, collectivism considers the degree to which people are integrated into various groups and examines their perceived obligations and dependence on groups [8]. In collectivist societies, greater importance is placed on the goals and well-being of the group as a whole [9]. The lens of collectivism has been used extensively to study a variety of issues including national wealth, employment, international relations and negotiations, with well-established conceptual frameworks such as the Hofstede, Trompenaars and GLOBE models [7,10,11].

A closely related concept to collectivism is interdependence, which describes the relationships and interactions of people and things, and is similarly embraced in other fields of study [12]. In social psychology, for example, interdependence theory states that all interpersonal relationships are defined by the interdependent interactions that influence one another’s experiences [13]. In quantum mechanics, nothing can be studied as separate from one another because all phenomena exist only in relation to the whole and to each other, and are thus, interdependent [14]. Where collectivism offers normative principles about what should be valued (e.g. belonging, service, unity), interdependence helps us understand how actions and outcomes are co-constructed through reciprocal engagement.

Collectivism and interdependence are particularly relevant to HPE, where identity, competence, and performance are shaped by ongoing interactions across teams, institutions, and broader healthcare systems. However, it is important to note that our goal is not to suggest the complete replacement of individualism, nor to ignore the value of personal agency, autonomy and accountability; rather, we aim to illuminate how a traditional reliance on individualist models may have constrained innovation and perpetuated inequity within HPE. To this end, our review study aims to address the following questions:

1. How have collectivism and interdependence been described and applied in healthcare and HPE literature?
2. What are the implications of embracing collectivism and interdependence within healthcare and HPE?

## Method

We conducted a narrative review (NR) to identify and synthesize literature about collectivism and interdependence to examine how these paradigms exist within the context of healthcare and HPE. NR was deemed an appropriate methodology because no work has previously examined in-depth the combination of collectivism and interdependence within the context of healthcare or HPE [15]. NR also offered the flexibility to select literature that could be consolidated, thereby building on relevant previous work without duplicating efforts [16]. Furthermore, our objective was not to systematically analyze or appraise the quality of currently available literature [17], but rather provide an overview of the current knowledge of collectivism and interdependence as it relates to healthcare and HPE. We followed the steps of NR methodology outlined in the literature as best practices [18,19,20].

### Initial search strategy

In January 2024, we searched the MEDLINE, Embase, PsycINFO and ERIC databases using the phrases “collectivism,” “interdependence,” and the following search strings: “collectivis* or interdependen*”, “training or education” and “medical or physician or health professional.” The final search string was: “((collectivis* OR interdependen*) AND (medical OR physician OR health professional)) OR ((collectivis* OR interdependen*) AND (training OR education))”. Initially, the ProQuest database was also included but, after consultation with a librarian, it was discarded as no articles identified during the initial search were found to be relevant to healthcare and HPE.

### Inclusion and exclusion criteria

We began by including publications written in English and published in peer-reviewed journals between 1990 and 2024. This 25-year time frame allowed us to capture the most recent literature about collectivism and interdependence, and we searched for work that spanned important shifts within healthcare and HPE. Inclusion criteria were: a) articles published in English, b) published in peer-reviewed journals, c) studies focusing on collectivism and/or interdependence, d) studies within the context of healthcare and/or HPE. We excluded articles that were not in English, perspective pieces, commentaries, or unrelated to healthcare or HPE.

### Screening, selection, and synthesis

**Figure 1:**
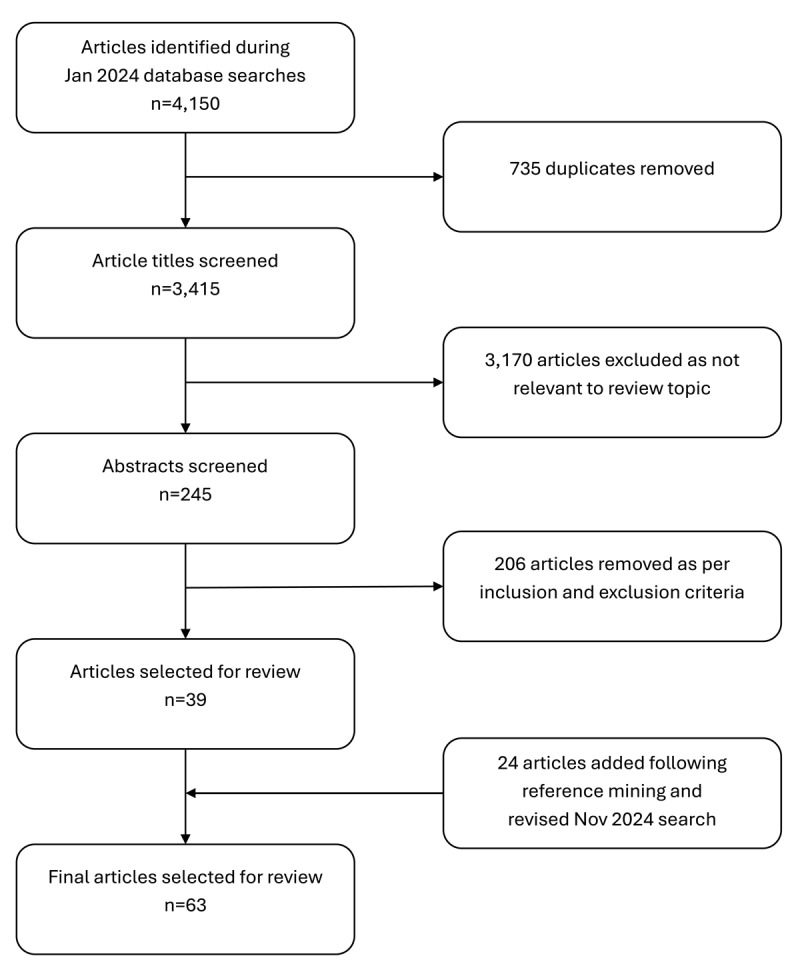
Article selection process for narrative review of the literature on the implications of collectivism and interdependence, 1990–2024.

Applying the inclusion and exclusion criteria, one author (EA) initially reviewed all titles and abstracts (n=3,145) to identify which articles should be selected for full-text screening. Two authors (EA and SSS) then conducted full-text screening (n=245), after which 39 articles were deemed relevant to the NR. Borderline papers were reviewed by the research team (EA, SSS, WKC, KE, JMM) who determined by consensus whether the papers met full criteria for inclusion. In November 2024, an additional 24 references were added through forward and backward reference mining. This led to a total of 63 articles that met final inclusion criteria (Figure 1).

We employed reflexive thematic analysis to consolidate the data [21]. This approach allowed for iterative and flexible exploration of patterns within the data, focusing on meaning-making rather than solely quantifying themes. Two authors (EA and SSS) independently familiarized themselves with the data and performed initial coding. Coding was conducted inductively, where themes were derived directly from the data, rather than using pre-determined theories or categorizations. Both authors then met regularly to discuss and refine the codes, identifying areas of agreement and divergence. Discrepancies were resolved through discussion, ensuring a shared understanding and interpretation of the data. All authors read the articles that met final inclusion criteria and participated in the thematic analysis to synthesize the literature into their relevant themes. Themes were developed collaboratively using a reflexive process that emphasizes the role of the researchers in actively interpreting the data.

### Reflexivity statement

The first author (EA) is an international medical graduate from South Korea, and two other authors (JMM & WJC) are also of Asian ethnic background, although predominantly raised in North America. SSS is a first-generation Canadian of Hungarian descent who currently resides in the United States and studies interdependence within medical education. KE is also Canadian and of German descent. JMM, WJC, SSS and KE were all raised in Canada. It should be noted that all authors have advanced education and training and currently practice in North America, an individualist cultural environment, yet their cultural backgrounds and perspectives are more collectivist in nature [11]. This diversity of the authors’ backgrounds and upbringings added to the richness of our interpretations of the literature.

From an epistemological perspective, all authors approached this review in a constructivist manner, seeking to find and make meaning of the disparate, seemingly disjointed literature in order to find a unifying narrative that offers new and valuable insights to healthcare and HPE [22]. In keeping with this epistemology, and the nature of a NR, the search methods are described to maintain transparency, rather than reproducibility [18].

## Results

Table 1 provides an overview of the references included in this review, including the original discipline and their respective thematic codes. Through reflexive thematic analysis, we identified four key, interrelated themes: (1) reshaping the culture of healthcare and the learning climate of HPE, (2) accounting for individual contributions to the team, (3) the many facets of leadership, and (4) belongingness as an essential step towards collective well-being (Table 2). Together these themes support a paradigm shift away from individualist assumptions, towards relational and interdependent models of education and patient care.

**Table 1:** Summary of articles (n = 63) included in narrative review, 1990–2024.


TITLE	AUTHOR	YEAR	TYPE	SOURCE	DISCIPLINE(S)	TOPIC(S)

A Dependent Structure of Interdependence: Structure and Agency in Relational Perspective	Crossley	2022	Article	Sociology	Sociology, Social psychology	Interdependence, Culture

A Scoping Review of Approaches for Measuring ‘Interdependent’ Collaborative Performances	Sebok-Syer, Shaw, Asghar et al	2021	Article	Medical Education	Health professions education	Interdependence, Assessment, Culture, Teamwork

A Theory of Collective Competence: Challenging the Neo-liberal Individualisation of Performance at Work	Boreham	2004	Article	British Journal of Educational Studies	Education, Teamwork	Collectivism, Assessment, Culture, Interdependence, Teamwork

Academic-Practice Partnerships: The Interdependence Between Leadership and Followership	Everett	2016	Article	Nursing Science Quarterly	Health professions education	Leadership, Followership, Teamwork, Interdependence

Awakening Compassion at Work: The Quiet Power That Elevates People and Organizations	Worline, Durron	2017	Book	Berrett-Koehler Publishers	Psychology	Compassion, Culture, Teamwork

Burnout, Mistreatment and Stress (in A Practical Guide for Medical Teachers)	Balaam, Harris	2021	Book	Elsevier Health Sciences	Health professions education	Compassion, Well-being

Can 40 Seconds of Compassion Reduce Patient Anxiety?	Fogarty, Curbow, Wingard et al	1999	Article	Journal of Clinical Oncology	Psychology, Economics	Compassion

Caring For Others Without Losing Yourself: An Adaptation of the Mindful Self-Compassion Program for Healthcare Communities	Neff, Knox, Gregory	2020	Article	Journal of Clinical Psychology	Psychology, Healthcare	Compassion, Well-being

Choosing Service Over Self-Interest	Block	1996	Book	Berrett-Koehler Publishers	Leadership, Management	Leadership, Interdependence, Collectivism, Culture

Collective Competence: Moving from Individual to Collaborative Expertise	Langlois	2020	Article	Perspectives on Medical Education	Health professions education	Teamwork, Collectivism, Assessment, Culture

Compassion and Efficiency Not Mutually Exclusive in Health Care	Brown	2019	Article	Canadian Medical Association Journal	Psychology, Economics	Compassion

Compassionomics: The Science and Practice of Caring	Lains, Johnson, Johnson	2024	Article	American Journal of Ophthalmology	Psychology, Economics	Compassion

Conceptualization of Jeong and Dynamics of Hwabyung	Chung, Cho	2006	Article	Psychiatry Investigation	Psychology	Collectivism, Culture

Considering the Interdependence of Clinical Performance: Implications for Assessment and Entrustment	Sebok-Syer, Chahine, Watling et al	2018	Article	Medical Education	Health professions education	Assessment, Interdependence, Teamwork

Coproducing Health Professions Education: A Prerequisite to Coproducing Health Care Services?	Englander, Holmboe, Batalden et al	2020	Article	Academic Medicine	Health professions education	Teamwork, Leadership, Culture

Cross-cultural Management	Abdelazim	2022	Article	Human Resource and Leadership Journal	Business, Management	Culture, Collectivism

Cultural Consequences: Comparing Values, Behaviours, Institutions, and Organizations across Nations	Hofstede	2001	Book	Sage Publications	Social psychology	Collectivism, Culture

Defining Cultural Competence: A Practical Framework for Addressing Racial/Ethnic Disparities in Health and Health Care	Betancourt, Green, Carillo et al	2003	Article	Public Health Reports	Sociology, Healthcare	Culture, Injustice

Dimensionalizing Cultures: The Hofstede Model in Context	Hofstede	2011	Article	Online Readings in Psychology and Culture	Social psychology	Collectivism, Culture

Distant and Hidden Figures: Foregrounding Patients in the Development, Content and Implementation of Entrustable Professional Activities	Sebok-Syer, Gingerich, Holmboe et al	2021	Article	Academic Medicine	Health professions education	Assessment, Interdependence

Effective Followership: A Standardized Algorithm to Resolve Clinical Conflicts and Improve Teamwork	Sculli, Fore, Sine et al	2015	Article	Journal of Healthcare Risk Management	Health professions education, Management	Followership, Teamwork, Leadership

Elucidating System-level Interdependence in Electronic Health Record data: What Are the Ramifications for Trainee Assessment?	Sebok-Syer, Pack, Shepherd et al	2020	Article	Medical Education	Health professions education	Assessment, Interdependence

Embracing the Collective Through Medical Education	Bleakley	2020	Article	Advances in Health Sciences Education	Health professions education	Collectivism, Culture

Embracing the Tension Between Vulnerability and Credibility: ‘Intellectual Candour’ in Health Professions Education	Molloy, Bearman	2019	Article	Medical Education	Health professions education	Culture

Emic Understandings of Attentiveness and its Related Concepts Among Japanese	Fukushima	2016	Article	East Asian Pragmatics	Psychology	Collectivism, Culture

Epistemic Solidarity in Medicine and Healthcare	Pot	2022	Article	Medicine, Health Care and Philosophy	Philosophy, Bioethics	Solidarity, Injustice

Equity, Diversity, and… Exclusion? A National Mixed Methods Study of “Belonging” in Canadian Undergraduate Medical Education	Sivananthajothy, Adel, Afhami et al	2023	Article	Advanced in Health Sciences Education	Health professions education	Well-being, Professional Identity, Collectivism, Injustice

Fitting in While Standing Out: Professional Identity Formation, Imposter Syndrome, and Burnout in Early-career Faculty Physicians	Vaa Stelling, Andersen, Suarez et al	2023	Article	Academic Medicine	Health professions education	Professional Identity, Well-being, Culture

Followership Theory: A Review and Research Agenda	Uhl-Bien, Riggio, Lowe et al	2014	Article	Leadership Quarterly	Leadership, Management	Leadership, Followership

Improving Knowledge and Behavior to Leadership and Followership Among the Interprofessional Team	Tschannen, Dron, Tedesco	2018	Article	International Journal of Medical Education	Health professions education	Teamwork, Followership, Leadership

Individualism-collectivism in Hofstede and GLOBE	Brewer, Venaik	2011	Article	Journal of International Business Studies	Management	Culture, Collectivism

Interdependence Theory (in APA Handbook of Personality and Social Psychology)	Van Lange, Balliet	2015	Book	American Psychological Association	Social psychology	Interdependence, Culture

Leadership and Followership Dynamics in Interprofessional Health Care Teams: Attending Physician Perspectives	Barry, Teunissen, Varpio et al	2024	Article	Academic Medicine	Health professions education, Military	Leadership, Followership, Teamwork

Leadership and Followership in Military Interprofessional Health Care Teams	Barry, Bader-Larsen, Meyer et al	2021	Article	Military Medicine	Leadership, Management	Leadership, Followership

Leadership and the New Science: Learning About Organization from an Orderly University	Wheatley	1992	Book	Berrett-Koehler Publishers	Leadership, Quantum physics	Interdependence, Leadership

Leadership for Change in the Education of Health Professionals	Neufeld	1995	Book	Network Publications	Leadership	Culture, Leadership, Teamwork

Learning the Value of Africa’s Collectivism for an Individualistic Europe	Modesti, Becucci	2019	Article	Internal and Emergency Medicine	Psychology	Culture, collectivism

Leveraging Electronic Health Record Data and Measuring Interdependence in the Era of Precision Education and Assessment	Sebok-Syer, Small, Lingard et al	2024	Article	Academic Medicine	Health professions education	Assessment, Interdependence

Maslow’s Hierarchy of Needs: Does it Apply in a Collectivist Culture?	Gambrel, Cianci	2003	Article	Journal of Applied Management and Entrepreneurship	Management	Collectivism, Culture

Medical “Heroes” and the COVID-19 Pandemic	Lipworth	2020	Article	Journal of Bioethical Inquiry	Bioethics	Leadership, Professional Identity

Paradoxical Truths and Persistent Myths: Reframing the Team Competence Conversation	Lingard	2016	Article	Journal of Continuing Education in the Health Professions	Health professions education	Teamwork, Assessment

Professionalism and the Socialization of Medical Students	Hafferty	2008	Book	Cambridge University Press	Health professions education	Professional Identity, Collectivism

Reluctant Heroes: New Doctors Negotiating Their Identities Dialogically on Social Media	Dornan, Armour, Bennett et al	2023	Article	Medical Education	Health professions education	Leadership, Professional Identity

Riding The Waves of Culture: Understanding Cultural Diversity in Global Business	Trompenaars, Hampden-Turner	1998	Book	McGraw Hill	Management, Social psychology	Culture, Collectivism

Shared Minds: The New Technologies of Collaboration	Schrage	1990	Book	Random House	Teamwork, Management	Leadership, Teamwork, Collectivism

Situated Learning Theory in Health Professions Education Research: A Scoping Review	O’Brien, Battista	2020	Article	Advances in Health Sciences Education	Health professions education	Professional Identity, Collectivism

Situated Learning: Legitimate Peripheral Participation	Lave, Wenger	1994	Book	Cambridge University Press	Education, Psychology	Collectivism, Professional Identity

Solidarity in Biomedicine and Beyond	Prainsack, Buyx	2017	Book	Cambridge University Press	Bioethics, Philosophy	Solidarity, Policy, Injustice

Solidarity in Public Health Ethics and Practice: Its Conceptions, Uses and Implications	Bellefleur, Keeling	2015	Article	National Collaborating Centre for Healthy Public Policy (Canada)	Health policy, Bioethics	Solidarity, Policy, Injustice

Solidarity in the Moral Imagination of Bioethics	Jennings, Dawson	2015	Article	Hastings Center Report	Bioethics, Philosophy	Solidarity

Supportive and Collaborative Interdependence: Distinguishing Residents’ Contributions Within Health Care Teams	Sebok-Syer, Lingard, Panza et al	2023	Article	Medical Education	Health professions education	Assessment, Interdependence, Teamwork

The 7 Habits of Highly Effective People: Powerful Lessons in Personal Change	Covey	2020	Book	Simon & Schuster	Leadership, Management	Culture, Interdependence

The Dangerous Pursuit of Independence	Regehr	2016	Abstract	The Centre for Health Education Scholarship, University of British Columbia	Health professions education	Interdependence, Collectivism, Culture

The Power of Followership: How to Create Leaders People Want to Follow, and Followers Who Lead Themselves	Kelley	1992	Book	Doubleday/Currency	Leadership, Management	Leadership, Teamwork, Followership

The Roseto Effect: A 50-year Comparison of Mortality Rates	Egolf, Lasker, Wolf et al	1992	Article	American Journal of Public Health	Epidemiology	Collectivism, Culture, Policy

The Wisdom of Teams: Creating the High-Performance Organization	Katzenbach, Smith	2006	Book	Harper Business Essentials	Management, Business	Teamwork, Leadership, Culture

The Wolf You Feed: Challenging Intraprofessional Workplace-based Education Norms	Stalmeijer, Varpio	2021	Article	Medical Education	Health professions education	Interdependence, Culture

Toward an Integrative View of Identity (in Handbook of Identity Theory and Research)	Vignoles, Schwartz, Luyckx	2011	Book	Springer	Psychology	Professional Identity, Collectivism

Toward Authentic Clinical Evaluation: Pitfalls in the Pursuit of Competency	Ginsburg, McIlroy, Oulanova et al	2010	Article	Academic Medicine	Health professions education	Assessment, Culture

Understanding Ubuntu and its Contribution to Social Work Education in Africa and Other Regions of the World	Mugumbate, Mpedziswa, Twikirize et al	2024	Article	Social Work Education	Education, Psychology	Collectivism, Culture

Unusually Low Incidence of Death From Myocardial Infarction: Study of an Italian American Community in Pennsylvania	Stout, Morrow, Brandt et al	1964	Article	The Journal of the American Medical Association	Epidemiology	Collectivism, Culture

What Does Solidarity Do for Bioethics?	Kolers	2021	Article	Journal of Medical Ethics	Bioethics, Philosophy	Solidarity

When I Say… Epistemic Injustice	Pervical, Martin, Maggio et al	2024	Article	Medical Education	Health professions education	Injustice, Solidarity


**Table 2:** Key themes identified through reflexive thematic analysis.


THEME	KEY INSIGHTS	REPRESENTATIVE CONCEPTS & FINDINGS

1. Reshaping the culture of healthcare and the learning climate of HPE	Culture is shaped by interactions, competitive vs collaborative dynamics, and the potential of solidarity.	Competitive medical school environments hinder teamwork. Siloed healthcare structures limit ‘collective responsibility.’ Communities of practice and interprofessional learning empower collective education. Solidarity (including epistemic solidarity) offers a collectivist counter to liberal individualism, fostering equity, reciprocity, and inclusive knowledge-sharing.

2. Accounting for individual contributions to the team	Performance and competence are relational, not individualistic. Teams thrive on interdependence and collective competence.	High-performing teams exceed expectations when members invest in each other’s growth. ‘Collective competence’ reframes assessment as distributed, contextual, and relational. Trainees inseparable from supervisors, systems, and patients → interdependence. Calls for assessment methods/language that capture team-based performance.

3. The many facets of leadership	Leadership is distributed, relational, and co-produced; followership and shared leadership are vital but constrained by hierarchy.	Shift away from the ‘heroic’ individual leader toward partnership and co-production. Followership is a necessary form of leadership. Shared leadership recognized but under-rewarded in hierarchical healthcare systems. Implications for redefining leadership in both healthcare and HPE.

4. Belongingness as an essential step towards collective well-being	Belonging and compassion underpin professional identity, well-being, and resilience.	Lack of belonging linked to imposter syndrome, burnout, depression (esp. underrepresented groups). Compassion (mindfulness, kindness, shared humanity) reduces stress and is cost-effective (‘compassionomics’). Professional identity shifts from ‘self’ → ‘self-in-relation’ → ‘self-in-profession.’ Socialization into the collective involves transformation of self, beyond skills/knowledge.


### 1. Reshaping the culture of healthcare and the learning climate of HPE

Several studies focused on aspects of the learning environment and how interactions among people and non-material aspects within a situation shaped culture. As an example, one reference noted that the competitive, rather than collaborative, environments of the medical school landscape make it difficult to create the “spirit of teamwork” [23]. This work characterized many healthcare learning environments as defensive, protective, and political, but argued that environments could be altered by focusing more on service, contributions and trust. Another reference noted how the siloed nature of healthcare workers inhibits our “collective responsibility” whereby individuals can have reciprocal vulnerability and trust, which can in turn transform learning environments [24]. Finally, one study found that fostering communities of practice as part of a culture of HPE within healthcare settings by using an interprofessional lens can better prepare medical trainees and empower teams to collectively support workplace-based education [25].

Through our reference mining, we discovered bioethics literature that discussed the concept of “solidarity,” defined as the commitment to accept costs to assist others and benefit the greater good [26]. Solidarity underpins several global and public health initiatives and can be used as a step towards addressing healthcare access and equity [27,28]. One study argued that the relative absence of solidarity in certain fields (e.g., healthcare and HPE) occurs because this collectivist value is at odds with the liberal individualism that prevails today [29]. Another article explored the concept of “epistemic solidarity”: the collective commitment to share, support and validate each other’s knowledge, experience and perspectives [30]. The authors highlight the potential of epistemic solidarity in fostering more equitable and collaborative environments, which in turn could benefit knowledge production and address some of the injustices rooted in existing healthcare and HPE structures [31]. This concept of epistemic solidarity aligns closely with the goals of fostering inclusive and supportive learning environments, where diverse perspectives are not only acknowledged, but actively integrated thereby creating spaces that encourage collaborative learning, mutual respect, and shared growth.

### 2. Accounting for individual contributions to the team

Many studies highlighted the power and impact of teams, citing evidence that a high-performing team “significantly outperforms all reasonable expectations given its membership” [32]. High-performing teams were found to be those composed of members that are deeply invested and committed to the personal growth and success of others within the team [32]. The phrase “collective competence” has been coined within HPE to reflect the changing discourse around competence. Collective competence refers to the distributed capacity of a system that is both contextual and relational [1]. Three studies spanning two decades advocated for a more collective approach that allows for holistic and authentic assessments within healthcare and affiliated educational settings [33,34,35].

Within the HPE assessment literature, several studies highlighted that medical trainees are often inseparable from their supervisors, the systems in which they work, and the patients they serve – a phenomenon that has been described in HPE as interdependence [36,37,38]. The most recent studies surrounding interdependence in HPE argue for measurement approaches [39] and assessment language [40] that more accurately reflect and account for the performance of individuals working within teams [33]. This emerging perspective within HPE supports a shift in focus from merely assessing individual performance to accounting for the interconnectedness between individual contributions and team dynamics, thereby emphasizing the importance of interdependence in fostering both individual and collective success.

### 3. The many facets of leadership

Some of the literature reviewed referred to the notion of partnership in relation to leadership, signaling an advancement in thinking beyond the patriarchal model that a single individual and their actions alone are responsible for the work of teams and communities [41]. As described by Englander, Lipworth, and Dornan, the concepts of partnership and co-production [42] challenge the stereotype of a singular “hero,” and redistribute power to more accurately characterize clinical and educational work that is done both intra- and inter-professionally [43,44]. These important concepts can reshape our conceptualizations of leaders and teams and have implications for both the practice of healthcare and HPE as a whole.

Along the lines of leadership, a few articles discussed the notion of “followership” as a specific form of leadership. The work of Uhl-Bien and Barry suggests that followership is a specific role and one that is necessary to enable leadership [45,46]. The work of Barry and colleagues has foregrounded the notion of shared leadership within our existing systems and structures [47]. The authors argue that shared leadership is critical in healthcare and HPE because of the shifting dynamics surrounding leadership within the clinical landscape. They also identified that despite shared leadership and followership being recognized and appreciated by individuals and groups, the possibilities for implementing this thinking within healthcare and HPE is often constrained due to hierarchies and systems that struggle to acknowledge or reward these actions.

### 4. Belongingness as an essential step towards collective well-being

Sivananthajothy and colleagues examined “belonging” in medical schools, particularly among those underrepresented in medicine (i.e., women, racialized minorities, Indigenous cultures, persons with disabilities, and 2SLGBTQIA+ peoples) [48]. They defined belonging as “a feeling of connectedness with social groups, physical places or experiences,” and found that a lack of belonging preceded imposter syndrome, negatively impacted learners’ well-being and career trajectory, and further exacerbated burnout and depression.

Another study argued that “compassion” within healthcare is essential to equip medical students and healthcare workers with the ability to “help one another to become well, stay well and to protect ourselves from stress and burnout” [49]. Attentiveness not only to oneself but also to others was noted and described as requiring a combination of mindfulness, self-kindness, and a sense of common humanity or shared suffering [50]. The field of “compassionomics” has demonstrated compassion to be a cost-effective approach [51,52,53]. In fact, one study showcased that compassionate rapport could be built in the oncology outpatient setting in a mere forty seconds and lead to a subsequent decrease in patients’ anxiety [54].

The literature also highlighted that it is not enough to have an identity that exists solely in the individual domain: “who am I?”, or even one that is relational: “who am I in relation to others?” A complete professional identity and sense of belonging exists within, and as a result of, the collective: “who am I in relation to the profession?” [55] One study argued the importance and implications of socialization into the collective by differentiating it from simple training: “while any occupational training involves learning new knowledge and skills, it is the melding of knowledge and skills with an altered sense of self that differentiates socialization from training” [56]. Differentiating oneself from mere knowledge and skills opens up the possibility of achieving a complete sense of self and well-being.

## Discussion

This review synthesized literature spanning three decades and identified four interconnected themes that illustrate how collectivism and interdependence function as alternative paradigms for understanding and transforming healthcare and HPE. These themes – reshaping culture, recognizing individual contributions within teams, reimagining leadership and followership, and fostering belongingness – serve as entry points to critically examine the dominance of individualism in educational structures and propose more relational alternatives reflective of the team-based reality of healthcare. At their core, these findings reflect misalignment between the values espoused in clinical practice and those embedded in HPE. While teamwork, collaboration and systems-based care are recognized as essential in contemporary healthcare delivery, educational models often continue to reward individual competition, autonomy, and performative leadership. The consequence is not only a pedagogical disconnect, but also a cultural and emotional strain on learners and educators alike. This dissonance calls attention to the importance of the cultural underpinning of how competence, identity and ultimately success are defined and assessed in HPE.

### Themes cultivated from the literature review

The first theme, reshaping culture and learning climate, reveals how competitive and hierarchical environments [4,5] are not without risk, including but not limited to stifling collaboration, decreased psychological safety, isolation and burnout [24]. The value of an interdependent culture lies in the fact that it can increase productivity through synergy, and create an environment that is safer and less judgmental, in exchange for vulnerability [23]. This is aligned with Molloy’s work on the benefits of fostering intellectual candor, where reciprocal vulnerability builds trust for truly transformative learning [57]. Collectivism and interdependence are also compatible with the partnership [41] and co-production [42] frameworks, whereby power is redistributed more evenly among individuals. With honesty as a prerequisite, partnership and co-production allow for co-creation by all, which in turn establishes joint accountability for successes and failures [41,42]. Finally, the framework of solidarity can be used to explore how both social and epistemic injustices can be addressed in healthcare and HPE [28,29,30,31]. These constructs can support a cultural paradigm shift that necessitates rethinking independence as the end-goal of training: it may not be the final destination, but rather a necessary step to achieving interdependence within the collective.

The second theme, recognizing individual contributions within teams, addresses the growing tension between individualized assessment models and team-based clinical reality. High-performing teams go beyond mere self-sacrifice or striving for what is collectively best [58]. Their success is rooted in the depth of team members’ commitment to one another’s personal growth and success [32]. Schrage makes the distinction between communication-oriented environments, where people “discuss what they want to do and then go off and do what they think they’ve agreed upon,” versus collaborative environments, where individuals “generate shared understandings that they couldn’t possibly have achieved on their own” [58]. Individualism indeed seems to run counter to the evidence base for effective interprofessional clinical teams [2]. This perspective does not imply that individuals and their contributions should be ignored. In fact, interdependence within teams advocates for a shift towards an assessment culture that captures individual contributions to the team as a means of recognizing and rewarding interdependent, collaborative behavior [36,40,59].

The third theme, reimagining leadership and followership, critiques the traditional, heroic model of leadership in medical education [43,44]. This model, which elevates singular decision-making and positional authority, is poorly suited to the complexity of healthcare systems that require shared governance and distributed expertise. Collectivism and interdependence suggest the importance of understanding and valuing followership, thereby recognizing that leaders and followers are often the same individuals with similar characteristics who assume different roles at different times throughout their clinical and educational careers [45,46,47,60,61]. Followership, in this view, is not passive but proactive; it involves questioning, complementing, and sustaining leadership efforts in ways that contribute to collective goals. Acknowledging and celebrating the dynamic and fluid nature of the teams involved in healthcare delivery, rather than fighting it, allows us to be creative in our approaches to patient care [62,63]. To be clear, we are not advocating for a diffusion of responsibility. Rather, we argue that interdependence is likely of maximal benefit when we strive to be the best we can possibly be as individuals while also working interdependently to help and collaborate with others as needed. Embracing interdependence involves a choice available to those with awareness of their independent abilities and understand the broader healthcare system, which in turn allows them to hold themselves to the highest standards of accountability.

The fourth theme, fostering belongingness, highlights the role of social connectedness in learner well-being, engagement, and identity formation. Both healthcare and HPE are social endeavors that cannot be accomplished in isolation, and thus we advocate for taking compassionate collective responsibility for one another in both settings. Compassionate systems foster work and learning environments where vulnerability is met with understanding, rather than persecution [53,64]. Often, medical teams with higher quality teamwork report higher rates of medical errors not because they make more mistakes, but because such teams have the psychological safety to report and learn from near-misses [65]. Without a sense of belonging and connectedness with the environment, experiences and people around us, the well-being of our healthcare workers, learners and educators will remain in permanent jeopardy [66]. Adrienne Rich once stated: “no person, trying to take responsibility for her or his identity, should have to be so alone. There must be those among whom we can sit down and weep and still be counted as warriors” [67]. Her words remind us that we do not have to be alone as we tackle the challenge of embracing a new identity for healthcare and HPE.

### Alignment with existing theories, frameworks, and cultural traditions

Taken together, these themes are interconnected and mutually reinforcing. Flattening traditional hierarchies strengthens shared leadership and belonging, as distributed decision making allows all team members to contribute meaningfully. This inclusive dynamic fosters trust, mutual respect, and shared ownership, deepening a collective sense of belonging within the learning environment.

These themes highlight the relevance of the conceptual frameworks that prioritize collective growth and interdependence. Frameworks such as Communities of Practice can be used to highlight how identity is shaped not just by personal experiences, but by collective engagement [68]. When individuals become part of a professional collective by transitioning from peripheral participation to full membership it enhances their sense of belonging [69]. Psychological safety and self-compassion frameworks can be used to connect burnout to the absence of a sense of belonging and interdependence within teams, thereby highlighting how collective support can mitigate stress [48,64]. Interdisciplinary Interdependence Theory, the interconnectedness of individuals within teams and larger systems, also provides a path forward [12]. Expanding on Interdependence Theory by applying it to the assessment of healthcare teams allows interdependence, which was once considered an assessment “problem,” to move towards identifying measures of individuals’ effective teamwork [40,59]. The enduring relevance of these theoretical frameworks underscores the imperative for educators to transcend existing paradigms and design curricular models that fully integrate and operationalize the principles of collectivism and interdependence.

Cultural traditions offer complementary perspectives that reinforce these paradigms. In Japan, where collectivism predominates [8,9,11], “those who arrive early to work tend to park farther away from their workplace in order to give way to those who arrive late” [70]. This is congruent with the Japanese practice of “omoiyari” [71], which underscores empathic consideration and attunement to the needs of others. This sense of collective belonging or inseparable interpersonal interdependence has been described through similar concepts in other cultures, such as “jeong” in Korea [72], which expresses enduring emotional bonds and mutual care. Similarly, “ubuntu” in Africa articulates a moral philosophy rooted in interconnectedness: “I am because we are” [73]. These paradigms of collectivism and interdependence are far-reaching and powerful, with known positive effects on even the physical health of those who embrace them [74,75]. They are more than anthropological curiosities; they reflect worldviews that center relational identity and shared humanity. While such concepts must be adapted thoughtfully, they offer powerful counterpoints to Western narratives of autonomy, control, and competition. Their relevance is arguably particularly salient in multicultural learning environments and in systems grappling with inequity, exclusion and moral fatigue.

### Implications and Recommendations

It is important to emphasize that collectivism and interdependence are not unqualified goods; they require careful application, especially in settings where conformity, loss of individual agency, or groupthink may pose risks. Moreover, not all learners, or educators, will have equal access to collective benefits unless systems are designed to ensure inclusion, voice, and justice. As such, any curricular shift must embed countermeasures (e.g., protected dissent, rotating roles, and explicit voice-safeguards) to protect against and mitigate such risk.

Embracing these paradigms is not about romanticizing togetherness, but about cultivating the conditions under which authentic collaboration, shared purpose and mutual respect can flourish. Given that a paradigm shift towards collectivism and interdependence may have already started, as evidenced by the number of references published within the past five years (n = 26), we ought to focus on understanding how to best leverage and harness these paradigms within healthcare and HPE.

### 1. Fostering relational teaching and learning environments

To begin, we need to foster collective practices and interdependent cultures within healthcare and HPE settings that promote psychological safety, encourage learners to seek feedback, express vulnerability without repercussion, and repair trust within healthcare and HPE. This includes training educators to model collaboration, interdependence, and intellectual candor in both clinical and teaching spaces. Encouraging genuine vulnerability and continuously modeling feedback among faculty to foster and role-model growth is imperative. Sometimes being an exemplar of vulnerability is enough for the next generation of learners to feel safe enough to do so themselves, but we must also take a stand and protect others as we work towards achieving this goal. Vulnerability alone, however, is often insufficient; institutions must actively protect those who take interpersonal risks, including learners who speak up, ask questions, or challenge dominant norms. Fostering trust requires not only modeling, but also structural safeguards that signal a shared commitment to safety and growth.

### 2. Reforming assessment systems to reflect interdependence

Secondly, we must construct assessment systems that are reflective of our health practices, incorporating assessments of not only individual or team competencies, but also recognizing and measuring individual contributions to team-based care that will move us towards more accurate and authentic assessments. This necessitates rethinking our approach to assessing individuals using more sophisticated models and carefully tracking how and what trainees are taught.

### 3. Reframing leadership and celebrating followership

Education systems must move beyond the binary of “leader” and “non-leader”, towards models of shared and situational leadership and find ways to reward and celebrate these approaches. One strategy would be to explicitly embed followership in curricula, to emphasize and reflect the realities of healthcare delivery and distributed leadership. When someone quietly contributes to the collective benefit of the team, it is important to recognize and value alternative leadership approaches publicly. If we engage in this practice often enough, the perceived value of followership and shared leadership can become commonplace.

### 4. Support context-sensitive research and policy change

Finally, we must support research on collectivism and interdependence to inform policy change and drive curricula by prioritizing collaboration and learner well-being. The conceptual and theoretical frameworks described above combined with the references listed in Table 1 provide a starting point for these efforts within healthcare and HPE settings.

These preliminary recommendations are not intended as quick and facile ways to facilitate a tectonic shift in paradigm towards collectivism and interdependence. Change is difficult and requires those with power to relinquish some sense of individual control to alter the status quo. Effective role-modelling of belonging starts with a sense of connectedness with our peers and environment that may not be always readily palpable when challenging deeply rooted social and cultural norms.

Real-world implementation, however, is likely to face considerable challenges. Accreditation bodies and licensing authorities may resist shifts that appear to dilute individual accountability, while institutional cultures steeped in hierarchy and competition may view collectivist practices as undermining established norms of excellence. Faculty and learners alike may struggle with reconciling vulnerability, shared leadership, and followership within systems that reward independence and individual accomplishment. Recognizing these barriers does not weaken the argument for collectivism and interdependence; rather, it underscores the importance of deliberate strategies that anticipate resistance, build alignment with regulatory structures, and support cultural change over time.

### Limitations

This NR is limited by the underrepresentation of the Global South in the literature [76], which is important because these are geographic areas where the cultural phenomena of collectivism or interdependence are likely to be more prevalent [11,77]. This likely signals that while searching for English-language literature, important publications in other languages may have been missed. Although full-text review and extraction was conducted by multiple authors (EA and SS), the initial title and abstract screening phase of the literature search was conducted by a single author (EA), which leaves open the possibility of bias in selection. As noted in our reflexivity statement, however, the authors are of a diverse cultural background, and we attempted to mitigate this through genuinely open-format discussion amongst authors when resolving disputes on borderline papers.

Finally, it should also be noted that this study did not fully consider paradigms of collectivism and interdependence with patients as important stakeholders and further study is warranted in this regard. More than just a limitation, exploring how to integrate patients as core members of the collective is a priority future research direction.

## Conclusion

Collectivism and interdependence provide a language and a logic for naming what many educators already feel but may struggle to articulate. That is, that learning is not a solo endeavor, that care is not delivered in isolation, and that professional identity is shaped in and through relationships with others. By embracing interdependence and collectivism as guiding principles within healthcare and HPE, healthcare professionals and educators can better prepare for the collaborative, team-based nature of healthcare. Our review and recommendations serve as a starting point for transforming healthcare and HPE in ways that meet the needs of increasingly interconnected and interdependent clinical and learning environments. Change is difficult, and leading change harder still, but a “heightened awareness of new and emerging themes may serve useful in the realities of reform” [78], which likely include collectivism and interdependence as important paradigms within healthcare and HPE.

## Artificial Intelligence

Used for reference/bibliography formatting.
